# Trends and disparities in alcohol-DWI license suspensions by suspension duration, North Carolina, 2007–2016

**DOI:** 10.1371/journal.pone.0310270

**Published:** 2024-09-20

**Authors:** Bhavna Singichetti, Yudan Chen Wang, Yvonne M. Golightly, Stephen W. Marshall, Rebecca B. Naumann

**Affiliations:** 1 Injury Prevention Research Center, The University of North Carolina at Chapel Hill, Chapel Hill, North Carolina, United States of America; 2 Department of Epidemiology, The University of North Carolina at Chapel Hill, Chapel Hill, North Carolina, United States of America; 3 Department of Counseling, North Carolina A&T State University, Greensboro, North Carolina, United States of America; 4 Department of Maternal and Child Health, The University of North Carolina at Chapel Hill, Chapel Hill, North Carolina, United States of America; 5 College of Allied Health Professions, University of Nebraska Medical Center, Omaha, Nebraska, United States of America; University of Idaho, UNITED STATES OF AMERICA

## Abstract

**Purpose:**

To examine trends and potential disparities in North Carolina (NC) driving while impaired by alcohol (alcohol-DWI) license suspensions from 2007–2016. Specific objectives included: 1) examining personal (e.g., race/ethnicity) and contextual (e.g., residential segregation) characteristics of alcohol-DWI license suspensions by suspension duration; and 2) examining trends in annual suspension rates by race/ethnicity, sex, and duration.

**Methods:**

We linked NC administrative licensing and county-level survey data from several sources from 2007–2016. Suspensions were categorized by duration: 1 to <4 years and 4 years or longer (proxies for initial and repeat suspensions, respectively). We calculated counts, percentages, and suspensions rates (per 1,000 person-years) with 95% confidence intervals, examined trends in annual suspension rates by race/ethnicity, sex, and suspension duration.

**Results:**

We identified 220,471 initial and 41,526 repeat license suspensions. Rates among males were three times that of females. 21-24-year-old (rates: 6.9 per 1,000 person-years for initial; 1.5 for repeat) and Black (4.1 for initial; 1.0 for repeat) individuals had the highest suspension rates. We observed decreases in annual initial and repeat suspension rates among males, but only in repeat suspensions for females during the study period. A substantial decrease in annual initial suspension rates was observed among Hispanic individuals relative to other racial/ethnic groups, while annual repeat suspension rates exhibited large decreases for most racial/ethnic groups. The highest overall suspension rates occurred in counties with higher proportions of the population without health insurance and with the highest levels of Black/White residential segregation.

**Conclusions:**

Potential disparities by race/ethnicity and sex existed by alcohol-DWI license suspension duration (i.e., initial vs. repeat suspensions) in NC. Contextual characteristics associated with suspensions, including a high degree of residential segregation, may provide indications of underlying structures and mechanisms driving potential disparities in alcohol-DWI outcomes.

## Introduction

Alcohol-impaired driving is a critical public health problem. In 2020 alone, alcohol-impaired crashes resulted in more than 11,000 fatal injuries in the US–a 14.3% increase from the previous year [1]. US estimates indicate that more than 9,000 fatal injuries could be prevented each year if drivers with a blood alcohol concentration (BAC) > 0.08 g/dL were kept off roads [2]. Related economic costs from alcohol-impaired driving crashes were estimated at approximately $44 billion USD in 2010 [1].

As with many public health problems, disparities by race, ethnicity, and sex in alcohol-impaired driving behaviors, crashes, and fatal and nonfatal injuries have long persisted; however, specific associations vary depending on the specific outcomes and data sources examined. Research on these disparities has primarily included either self-report data to examine impaired driving behaviors and crash data to examine impaired driving-related crashes and corresponding injuries. Prior research using both self-report and crash data indicate that rates of alcohol-impaired driving and fatal crashes are consistently higher in males than females [1, 3–9]. However, research on racial and ethnic differences has been less consistent. Jewett et al (2015) [10] observed that percentages of adults reporting alcohol-impaired driving were similar between non-Hispanic White, non-Hispanic Black, Hispanic, and non-Hispanic multiracial subgroups–all of which were greater than a non-Hispanic Other subgroup. Meanwhile, other studies utilizing self-report data observed that White individuals had greater risks of alcohol-involved driving behavior, compared to Black, Hispanic, and Other groups [5, 11, 12]. Analyses of drivers as well as other road users (e.g., passengers, pedestrians) in crash data observed that Hispanics and American Indians have the highest rates of involvement in fatal impaired driving-related crashes, while Asians have the lowest rates of involvement [12–15]; however, most analyses are several years old, requiring updated examination of trends.

Enforcement or arrest data provides a third and critical additional perspective into understanding alcohol-impaired driving and related negative outcomes, by offering an assessment of how often drivers are cited for impaired driving behaviors and providing insight on potential disparities in enforcement practices, particularly when considered within the context of other impaired driving data sources and estimates. Limited prior research utilizing enforcement data indicates that impaired driving arrest rates are higher in males than in females [7] and that Hispanics and American Indians may be overrepresented in alcohol-related driving offenses [12]. Additionally, research shows significant disparities in overall traffic stop data, albeit not alcohol-impaired driving stops specifically, with one study finding that although Black drivers spent less time driving and were less likely to have contraband in their vehicles, they were more likely to be stopped and more likely to have their vehicles searched compared to White drivers [16].

Household income, employment status, and/or highest level of education are often used as indicators of socioeconomic status (SES) in epidemiologic research, including in studies of alcohol-use and trends in alcohol-involved driving [5, 11, 17–19]. Identified associations between these indicators of SES and alcohol-involved driving have varied in studies, however, with some studies finding low levels of education, unemployment, and/or low income associated with higher levels of alcohol-involved driving behavior, while others observed associations between higher education and/or family/personal income with this behavior [5, 11, 19]. Additionally, characteristics of and factors related to geographic location, such as availability of alcohol, social norms, availability of treatment/rehabilitation programs, and policing resources and practices [20–24], are known to influence alcohol-involved driving, alcohol-DWI arrests and convictions, and alcohol-involved motor vehicle crashes, and often vary considerably across categories of urban/rural status and residential segregation. Urban/rural status has been regularly evaluated in alcohol use and alcohol-DWI research [5, 20, 25–28]. Residential segregation is used in a diverse range of research as a proxy for structural racism and is known to be associated with key public health outcomes and adverse environmental factors, including poor overall health, poor access to essential services like health care, alcohol outlet overconcentration, and disparities in SES [29–36].

Understanding trends in alcohol-impaired driving from multiple perspectives is crucial. Drivers’ license suspensions–a direct consequence of driving while impaired by alcohol (alcohol-DWI) citations–are especially important to examine. Several studies have indicated that license suspensions are a more effective strategy for reducing rates of alcohol-DWI and alcohol-related crashes, compared to use of jail terms, fines, and alcohol education programs only [37–41]. However, licenses are also recognized by the US Supreme Court to be ‘essential in the pursuit of a livelihood’ and can have negative implications for one’s employment, access to healthcare, and livelihood [42]. Research is lacking on a more holistic understanding of the context surrounding potential disparities in alcohol-impaired driving behaviors and license-related outcomes, including an understanding of the social and environmental context underlying these outcomes (e.g., economic instability, access to healthcare).

Thus, the objectives of this paper were two-fold: 1) to examine differences and disparities in personal and contextual (including structural social determinants of health) characteristics of alcohol-DWI license suspensions by suspension duration; and 2) to examine trends in the annual rates of these suspensions from 2007–2016. We examined data from one large state (North Carolina [NC]), where there were over 26,000 alcohol-DWI convictions imposed in Fiscal Year 2022 alone (July 1, 2021 through June 30, 2022) and an annual alcohol-DWI fatality rate that consistently sits higher than the national average [3, 43]. While analyses focused on one state, this study provides an analytic framework and describes important data system recommendations, particularly for improving assessment of disparities, that are applicable to other states across the U.S.

## Methods

### Study design and data sources

This study included drivers ages 21 to 64 years old with alcohol-DWI license suspensions occurring between 2007 and 2016, as determined from licensing data from the NC Department of Motor Vehicles (DMV). This age range was used as alcohol-impairment laws differ for individuals under the age of 21 and as license renewal policies often change for older adults. Further, older drivers often alter, restrict or eliminate driving. Those who are no longer driving are no longer at risk for an alcohol-DWI license suspension. While there is no one age at which older adults change their driving patterns, generally research defines older adults as age 65 and older, which we have adopted here [44]. NC licensing data includes records of all individuals who successfully obtain a driver’s license in NC and/or have a license-related event, such as a DWI license suspension.

Additional contextual data surrounding individuals with alcohol-DWI license suspensions were obtained from the following publicly available sources: American Community Survey (ACS) 5-year estimate tables, County Health Rankings (CHR) annual data, and Rural-Urban Continuum Codes (RUCC). Contextual variables were linked to suspensions by county of driver residence and suspension start year. The ACS is a national annual survey administered by the US Census Bureau that includes detailed county-level estimates of social, economic, housing, and demographic characteristics of communities [45–47]. The CHR program collects data from diverse data sources (e.g., Behavioral Risk Factor Surveillance System, the National Center for Health Statistics, the American Medical Association) and standardizes county-level measures that reflect population health [48, 49]. Finally, RUCC classifications developed by the Economic Research Service at the US Department of Agriculture, place counties into one of nine metropolitan and non-metropolitan categories based on population and commuter criteria [50, 51].

Licensing data used in this study were obtained by the University of North Carolina at Chapel Hill (UNC-Chapel Hill) from the NC DMV for research purposes on April 25^th^, 2019, following review and approval by the Institutional Review Board (IRB) at UNC-Chapel Hill (IRB #17–2906) and a signed Data Use Agreement (executed April 12, 2019). The current study used data collected under the aforementioned approvals, and therefore the study design and secondary analytic plan were reviewed and approved by the IRB at UNC-Chapel Hill (IRB #22–0270) as an extension of the previously approved IRB application (IRB #17–2906). Only authors conducting analyses had access to identifying information in this retrospective data; however, identifiers were removed prior to analyses.

### Measures

We examined trends and potential disparities in continuous alcohol-DWI license suspension events and the personal and contextual characteristics surrounding these events.

#### Alcohol-DWI license suspension events

Alcohol-DWI license suspensions were first identified from NC licensing records based on a pre-existing flag variable for all alcohol-related events. NC licensing data can include multiple records for a single alcohol-impaired driving event (i.e., civil revocations versus post-conviction suspensions); however, details linking multiple records to a single impaired driving event were not available. For this study, we combined records occurring for the same individual with overlapping start and end dates as continuous alcohol-DWI suspension events to capture continuous periods of suspension experienced by the individual.

We categorized continuous alcohol-DWI license suspension events by their duration, which was calculated using the start and end dates for each continuous suspension event. Categories included: 1-year to less than 4-year suspensions (proxy for initial suspensions), and 4-year or longer suspensions (proxy for repeat suspensions). These suspension type categories were developed based on suspension durations outlined in NC DWI conviction policies [52, 53]. Briefly, if convicted of an alcohol-DWI for the first time in 3 years, individuals will obtain a 1-year suspension. If an individual obtains another alcohol-DWI suspension within 3 years, they will obtain a 4-year suspension. Within each of these categories, the actual duration of a license suspension can vary based on several factors, including when the NC DMV receives an individual’s certificate of completion and when the requisite suspension period has expired. Thus, while most suspension events are 1 year (for an initial suspension) or 4 years (for a repeat suspension), some events last longer due to delay in receipt of the certificate of completion by the NC DMV. Certificates of completion, which confirm that the individual has completed all activities as required by NC legislation (e.g., substance abuse assessment), are a requirement for reinstating one’s license [54]. Since categorization in this study was determined solely on duration, the population at risk for ‘repeat’ suspension was not conditioned on having an ‘initial’ suspension. Continuous suspension events lasting less than 1 year, not included in this analysis, may be indicative of individuals who are suspected of impaired driving, as those who have BAC levels above legal limits (0.08 g/dL in NC) receive a civil revocation (pre-conviction/administrative) license suspension of at least 30 days. A longer duration category (i.e., an initial or repeat suspension type) nearly always come after these shortened suspension events (administrative license suspensions) based on NC DWI policies, albeit after a gap due to legal processes and court loads.

#### Personal characteristics of drivers with alcohol-DWI license suspension events

To examine differences and potential disparities in the events experienced by drivers, race/ethnicity and sex for each individual driver was obtained from licensing data. Both variables were collected via self-report at time of licensure or when a change in license information was conducted. Race/ethnicity, an optional variable not required for obtaining licensure, consisted of the following 5 categories: Asian, Black, Hispanic, American Indian, and White. While optional, missingness was low at less than 2.0%. For sex, individuals could select either ‘Female’ or ‘Male’ (less than 0.01% missingness). Age at suspension was calculated based on date of birth and date of license suspension and categorized into age groups. Date of birth was a required variable collected at time of licensure, and date of suspension was a required variable any time a license suspension occurred and was added to the NC licensing database.

#### Contextual characteristics associated with drivers having alcohol-DWI license suspension events

Contextual variables included several county-level measures that were matched to suspensions using an individual’s county of residence from licensing data. A four-level urban/rural variable was created by collapsing the original 9 RUCC classifications into the following categories: counties in metro areas (Metro), non-metropolitan counties with an urban population of 20,000 or more (Urban 20K or more), non-metropolitan counties with an urban population of 2,500 to 19,999 (Urban 2.5K -19,999), and counties that are completely rural or less than 2,500 urban population (Rural). Additional contextual variables included percent of the county population without health insurance, living below the poverty level in the last 12 months, and unemployed from the ACS, as well as the ratio of population to primary care physicians, percent of the county population reporting excessive (heavy or binge) drinking, and a residential segregation index from CHR data. The Black/White residential segregation index measures the degree to which Black and White residents in a county live separately from one another, with scores ranging from 0 for complete integration to 100 for complete segregation. These variables were selected based on prior research and team expertise regarding factors considered important for examining potential underlying structures driving potential disparities in alcohol-impaired driving events from a larger social determinant of health and socio-ecologic perspective [55].

In addition to being matched by county, all contextual variables except urban/rural status were matched to suspensions by year, when available. For years where a contextual data variable was not available, available data that was closest in time to that year was used. For example, data for percent uninsured and percent below poverty were only available from 2012 to 2016, so for suspensions starting between 2007 and 2011, contextual values from 2012 were used. Given the time it takes for notable changes in these variables to occur, using data from the nearest date should represent a reasonable approximation. For each variable, an annual average was calculated for each of the 100 NC counties across the study period, 2007–2016. We then classified counties into quartiles for each variable. Finally, urban/rural county classifications were matched to suspensions in two groups: 2003 RUCC classifications were used for suspensions that started between 2007 and 2012, and 2013 RUCC classifications were used for suspensions that started between 2013 and 2016.

### Analyses

We examined the distribution of continuous alcohol-DWI license suspensions in NC from 2007–2016 by race/ethnicity and sex, as well as by other key personal and contextual characteristics, stratified by license suspension type (i.e., initial vs. repeat). We calculated counts (number of continuous suspension events), percentages, and rates of continuous suspension events per 1,000 person-years, along with 95% confidence intervals (CIs). Population denominators for calculating rates (overall, by sex, by race/ethnicity, by county) were obtained from US Census Bureau estimates produced by the National Center for Health Statistics and the NC Office of State Budget and Management [56–58]. A complete case analysis was used for this study as missing data was minimal (i.e., <4% for all variables).

Next, we examined trends over time in alcohol-DWI license suspension rates by sex and race/ethnicity, stratified by suspension type. We also conducted a secondary analysis to examine the precise duration of each suspension event (i.e., examining license suspension-years from a continuous standpoint, rather than categorical standpoint of number of events) to specifically examine extended suspension durations due to delays in ending a suspension (e.g., inability to complete a substance use disorder assessment). To do this, we analyzed the specific lengths of suspensions by calculating the total number of years suspended, percent of years suspended, and rates of suspension-years per 1,000 person-years with 95% CIs, overall and by suspension type. As our data included a census of all alcohol-DWI license suspension events in NC and due to best practice guidance on moving away from a reliance on p-values to examine differences, we examined and described the overall magnitude and differences in the percentages and rates (with CIs) of personal and contextual characteristics, and in trends, by suspension type [59]. All rate estimates and CIs are presented to two significant figures.

## Results

### Personal characteristics of drivers with alcohol-DWI license suspension events

Between 2007 and 2016, there were 261,997 total continuous alcohol-DWI suspension events in NC lasting 1 year or longer among individuals ages 21 to 64 years old (Table 1). This included 220,471 with suspension durations of 1 year to <4 years (proxy for initial suspensions) and 41,526 with suspension durations of 4 years or longer (proxy for repeat suspensions). We present overall results on all continuous alcohol-DWI suspension events lasting 1 year or longer in the state from 2007–2016, and then specifically compare differences in initial and repeat suspensions.

**Table 1:** Personal characteristics of drivers* with alcohol-DWI suspensions in North Carolina, 2007–2016.

	Total Suspension Events			Suspension Duration 1 year to <4 years			Suspension Duration 4 years or longer		
				(proxy for initial suspension)			(proxy for repeat suspension)		
	Total no. of suspension events	% of suspension events	Rate of suspension events per 1,000 person-years (95% CI)	Total no. of suspension events	% of suspension events	Rate of suspension events per 1,000 person-years (95% CI)	Total no. of suspension events	% of suspension events	Rate of suspension events per 1,000 person-years (95% CI)
**Total**	261,997	-	4.6 (4.6, 4.6)	220,471	84.2**	3.9 (3.9, 3.9)	41,526	15.8**	0.73 (0.73, 0.74)
**Sex**									
** Female**	64,894	24.8	2.2 (2.2, 2.3)	55,867	25.3	1.9 (1.9, 1.9)	9,027	21.7	0.31 (0.31, 0.32)
** Male**	197,080	75.2	7.1 (7.1, 7.1)	164,589	74.7	5.9 (5.9, 6.0)	32,491	78.3	1.2 (1.2, 1.2)
**Race/ethnicity**									
** Asian**	1,547	0.6	1.0 (0.93, 1.0)	1,401	0.6	0.89 (0.84, 0.94)	146	0.4	0.09 (0.08, 0.11)
** Black**	63,083	24.6	5.1 (5.1, 5.2)	50,783	23.5	4.1 (4.1, 4.2)	12,300	30.1	1.0 (1.0, 1.0)
** Hispanic**	17,245	6.7	3.9 (3.9, 4.0)	14,782	6.8	3.4 (3.3, 3.4)	2,463	6.0	0.56 (0.54, 0.58)
** American Indian**	3,187	1.2	4.6 (4.5, 4.8)	2,626	1.2	3.8 (3.7, 4.0)	561	1.4	0.82 (0.75, 0.89)
** White**	171,865	66.9	4.6 (4.5, 4.6)	146,480	67.8	3.9 (3.9, 3.9)	25,385	62.1	0.67 (0.67, 0.68)
**Age at suspension**									
** 21–24**	45,543	17.4	8.4 (8.3, 8.5)	37,379	17.0	6.9 (6.8, 7.0)	8,164	19.7	1.5 (1.5, 1.5)
** 25–34**	89,472	34.2	7.1 (7.0, 7.1)	74,408	33.7	5.9 (5.8, 5.9)	15,064	36.3	1.2 (1.2, 1.2)
** 35–44**	61,564	23.5	4.7 (4.6, 4.7)	51,541	23.4	3.9 (3.9, 3.9)	10,023	24.1	0.76 (0.75, 0.78)
** 45–54**	46,740	17.8	3.4 (3.4, 3.5)	40,260	18.3	3.0 (2.9, 3.0)	6,480	15.6	0.48 (0.46, 0.49)
** 55–64**	18,678	7.1	1.6 (1.6, 1.6)	16,883	7.7	1.4 (1.4, 1.5)	1,795	4.3	0.15 (0.15, 0.16)

* Note: Drivers may appear more than once in the table if they had multiple suspension events during the study period.

**Percent of total suspensions

Sex–based on self-report and required for licensing data; missingness <0.01%

Race/ethnicity–based on self-report where individuals may select one designation and optional for licensing data; missingness = 1.94%

Age at suspension–calculated using date of birth (required for licensing data) and start date of suspension event; missingness = 0.00%

The number and rate of suspension events per 1,000 person-years among males was approximately three times that of females for initial events (male vs female rates: 5.9 vs 1.9), and among repeat suspensions, this difference was even greater (1.2 among males vs. 0.31 among females). The highest rates of suspension events occurred among Black individuals for both initial and repeat suspensions (initial: 4.1 per 1,000 person-years (95% CI: 4.1, 4.2); repeat: 1.0 (95% CI: 1.0, 1.0)). Among initial suspensions, White individuals had the second highest rate of suspension events (3.9; 95% CI: 3.9, 3.9), followed closely by American Indian (3.8; 95% CI: 3.7, 4.1) and Hispanic individuals (3.4; 95% CI: 3.3, 3.4). Among repeat suspensions, American Indian individuals had the second highest rate (0.82; 95% CI: 0.75, 0.89), followed closely by White (0.67; 95% CI: 0.67, 0.68) and Hispanic (0.56; 95% CI: 0.54, 0.58) individuals. Asian individuals consistently had the lowest rates of suspensions. Young adults (21–24-year-olds) demonstrated the highest rates of both initial and repeat suspensions (initial: 6.9 per 1,000 person-years [95% CI: 6.8, 7.0]; repeat: 1.5 [95% CI: 1.5, 1.5]), with rates 5–8 times that of 55–64-year-olds (initial: 1.4 per 1,000 person-years [95% CI: 1.4, 1.5]; repeat: 0.15 [95% CI: 0.15, 0.16]).

The secondary analysis of suspension-years (S1 Table), as compared to suspension events, indicated similar patterns. The rate of suspension-years per 1,000 person-years for initial suspension events among males was about 3 times that of females (male rate: 8.3 per 1,000 person-years [95% CI: 8.2, 8.3]; female rate: 2.6 [95% CI: 2.6, 2.7]), and for repeat events, was almost 4 times that of females (male rate: 7.0 [95% CI: 7.0, 7.1]; female rate: 1.8 [95% CI: 1.8, 1.8]), indicating similar relative differences in durations served. However, for race/ethnicity, rates of suspension-years displayed wider ranges, indicating potential disparities in the actual duration of suspension events served. The rate of initial *suspension events* for Black individuals was 4.5 times higher than Asian individuals, the subgroup with the lowest rates (Black rate: 4.1 suspensions per 1,000 person-years [95% CI: 4.1, 4.2]; Asian rate: 0.89 [95% CI: 0.84,0.94]); whereas the rate of initial *suspension-years* served was 5 times higher among Black individuals than Asian individuals (Black rate: 6.1 suspension-years per 1,000 person-years [95% CI: 6.0, 6.1]; Asian rate: 1.2 [95% CI: 1.1, 1.2]). Similarly, for repeat suspensions, the rate of suspension events was 10 times higher among Black individuals (rate: 1.0 initial suspension events per 1,000 person-years; 95% CI: 1.0, 1.0), as compared to Asian individuals (rate: 0.09; 95% CI: 0.08, 0.11), while the rate of suspension-years was 12.4 times higher (Black rate: 6.2 suspension-years per 1,000 person-years [95% CI: 6.1, 6.2]; Asian rate: 0.53 [95% CI: 0.49, 0.56]). For initial suspensions, Black individuals completed an average of 1.5 years per suspension event; American Indian and White individuals an average of 1.4 years; and Hispanic and Asian individuals an average of 1.3 years. For repeat suspensions, Black individuals completed an average of 6.2 years per suspension event; American Indian individuals an average of 6.1 years; White and Hispanic individuals an average of 5.8 years; and Asian individuals an average of 5.7 years.

### Contextual characteristics associated with drivers having alcohol-DWI license suspension events

Findings from analyses of contextual variables are presented in Table 2 and S2 Table. For both initial and repeat suspensions, metro counties had the largest percentage of suspension events (initial: 73.0%; repeat: 70.5%); however, urban counties with populations of 20,000 or more had the highest rate of suspensions (initial: 4.2 suspension events per 1,000 person-years [95% CI: 4.1, 4.2]; repeat: 0.85 [95% CI: 0.83, 0.87]). The largest rate of both initial and repeat alcohol-DWI license suspension events occurred in counties in the highest quartile of population uninsured (initial: 4.2 suspension events per 1,000 person-years [95% CI: 4.2, 4.3]; repeat: 0.85 [95% CI: 0.83, 0.88]). There was little variation in rates of overall suspension events by quartile of unemployment or proportion of the population living below the poverty limit (range: 4.4 to 4.7).

**Table 2:** Contextual/county-level characteristics of drivers with alcohol-DWI suspensions in North Carolina, 2007–2016.

	Total Suspension Events			Suspension Duration 1 year to <4 years			Suspension Duration 4 years or longer		
				(proxy for initial suspension)			(proxy for repeat suspension)		
	Total no. of suspension events	% of suspension events	Rate of suspension events per 1,000 person-years (95% CI)	Total no. of suspension events	% of suspension events	Rate of suspension events per 1,000 person-years (95% CI)	Total no. of suspension events	% of suspension events	Rate of suspension events per 1,000 person-years (95% CI)
**Urban/rural status**									
** Metro area**	186,044	72.6	4.4 (4.4, 4.4)	157,909	73.0	3.8 (3.7, 3.8)	28,135	70.5	0.67 (0.66, 0.68)
** Urban area with a pop of 20,000 +**	41,562	16.2	5.0 (5.0, 5.1)	34,511	15.9	4.2 (4.1, 4.2)	7,051	17.7	0.85 (0.83, 0.87)
** Urban area with a pop of 2,500–19,999**	21,049	8.2	4.7 (4.6, 4.7)	17,622	8.1	3.9 (3.9, 4.0)	3,427	8.6	0.76 (0.74, 0.79)
** Rural area**	7,653	3.0	4.6 (4.5, 4.7)	6,362	2.9	3.8 (3.7, 3.9)	1,291	3.2	0.78 (0.74, 0.82)
**% uninsured (median county-level uninsured percentage: 15.7%)**									
** Quartile 1: < 14.5%**	73,919	28.8	4.1 (4.1, 4.2)	62,957	29.1	3.5 (3.5, 3.5)	10,962	27.5	0.61 (0.60, 0.62)
** Quartile 2: 14.5% to <15.7%**	87,858	34.3	4.9 (4.9, 5.0)	73,755	34.1	4.1 (4.1, 4.2)	14,103	35.3	0.79 (0.78, 0.81)
** Quartile 3: 15.7% to <17.4%**	68,387	26.7	4.4 (4.4, 4.4)	57,942	26.8	3.7 (3.7, 3.8)	10,445	26.2	0.67 (0.66, 0.68)
** Quartile 4: ≥ 17.4%**	26,144	10.2	5.1 (5.0, 5.1)	21,750	10.1	4.2 (4.2, 4.3)	4,394	11.0	0.85 (0.83, 0.88)
**% unemployed (median county-level unemployed percentage: 10.7%)**									
** Quartile 1: < 9.1%**	81,833	31.9	4.6 (4.5, 4.6)	69,932	32.3	3.9 (3.9, 3.9)	11,901	29.8	0.66 (0.65, 0.68)
** Quartile 2: 9.1% to <10.7%**	79,255	30.9	4.4 (4.3, 4.4)	67,400	31.1	3.7 (3.7, 3.7)	11,855	29.7	0.65 (0.64, 0.67)
** Quartile 3: 10.7% to <11.9%**	55,136	21.5	4.7 (4.6, 4.7)	45,935	21.2	3.9 (3.9, 3.9)	9,201	23.1	0.78 (0.77, 0.80)
** Quartile 4: ≥ 11.9%**	40,084	15.6	4.7 (4.6, 4.7)	33,137	15.3	3.8 (3.8, 3.9)	6,947	17.4	0.81 (0.79, 0.83)
**% living below poverty limit (median county-level poverty percentage: 18.6%)**									
** Quartile 1: < 16.0%**	109,763	42.8	4.4 (4.3, 4.4)	93,930	43.4	3.7 (3.7, 3.7)	15,833	39.7	0.63 (0.62, 0.64)
** Quartile 2: 16.0% to <18.6%**	79,629	31.1	4.7 (4.7, 4.7)	66,849	30.9	3.9 (3.9, 4.0)	12,780	32.0	0.75 (0.74, 0.77)
** Quartile 3: 18.6% to <22.6%**	34,779	13.6	4.7 (4.6, 4.7)	29,000	13.4	3.9 (3.8, 3.9)	5,779	14.5	0.78 (0.76, 0.80)
** Quartile 4: ≥ 22.6%**	32,137	12.5	4.7 (4.7, 4.8)	26,625	12.3	3.9 (3.9, 4.0)	5,512	13.8	0.81 (0.79, 0.83)
**Health care access (Ratio of population to primary care providers; median ratio: 1,988.4)**									
** Quartile 1: < 1,347.4**	124,124	48.4	4.3 (4.3, 4.3)	10,5695	48.8	3.7 (3.6, 3.7)	18,429	46.2	0.64 (0.63, 0.65)
** Quartile 2: 1,347.4 to <1,988.4**	55,901	21.8	4.8 (4.8, 4.9)	46,878	21.7	4.1 (4.0, 4.1)	9,023	22.6	0.78 (0.77, 0.80)
** Quartile 3: 1,988.4 to <2,951.0**	46,104	18.0	4.8 (4.8, 4.9)	38,436	17.8	4.0 (4.0, 4.1)	7,668	19.2	0.80 (0.79, 0.82)
** Quartile 4: ≥ 2,951.0**	30,179	11.8	4.6 (4.6, 4.7)	25,395	11.7	3.9 (3.9, 3.9)	4,784	12.0	0.73 (0.71, 0.76)
**Excessive drinking (median county-level percentage: 12.0%)**									
** Quartile 1: < 9.9%**	27,161	10.6	4.8 (4.7, 4.8)	22,539	10.4	4.0 (3.9, 4.0)	4,622	11.6	0.81 (0.79, 0.84)
** Quartile 2: 9.9% to <12.0%**	35,490	13.8	4.9 (4.8, 4.9)	29,615	13.7	4.1 (4.0, 4.1)	5,875	14.7	0.80 (0.78, 0.82)
** Quartile 3: 12.0% to <14.0%**	75,458	29.4	4.6 (4.6, 4.6)	63,346	29.3	3.9 (3.8, 3.9)	12,112	30.4	0.74 (0.72, 0.75)
** Quartile 4: ≥ 14.0%**	118,199	46.1	4.4 (4.4, 4.4)	100,904	46.6	3.7 (3.7, 3.8)	17,295	43.3	0.64 (0.63, 0.65)
**Residential segregation index (median county-level index: 36)**									
** Quartile 1: < 28**	29,493	11.7	4.4 (4.4, 4.5)	24,809	11.7	3.7 (3.7, 3.8)	4684	11.9	0.70 (0.68, 0.72)
** Quartile 2: 28 to <36**	37,357	14.8	4.4 (4.4, 4.5)	31,115	14.6	3.7 (3.7, 3.7)	6242	15.9	0.74 (0.73, 0.76)
** Quartile 3: 36 to <46**	79,232	31.4	4.3 (4.3, 4.4)	67,289	31.6	3.7 (3.7, 3.7)	11943	30.4	0.65 (0.64, 0.67)
** Quartile 4: ≥ 46**	106,088	42.1	4.8 (4.7, 4.8)	89,693	42.1	4.0 (4.0, 4.1)	16395	41.8	0.74 (0.73, 0.75)

Urban/Rural–from Rural-Urban Continuum Codes (RUCC); missingness = 2.02% due to missing county information in suspension records

Uninsured, Unemployed, Poverty–from American Community Survey (ACS); missingness = 2.02% due to missing county information in suspension records

Health care access, Excessive drinking–from County Health Rankings (CHR); missingness = 2.02% due to missing county information in suspension records

Racial segregation–from County Health Rankings (CHR); missingness = 3.51% due to missing county information in suspension records and/or no residential segregation index score in CHR

Example interpretation for contextual variables: 28.8% of alcohol-DWI license suspension events occurred in counties falling in the lowest quartile of population uninsured, with an alcohol-DWI license suspension rate in these counties of 4.1 per 1,000 person-years.

Among both initial and repeat suspensions, almost half (initial: 46.6%; repeat: 43.3%) occurred in the counties within the highest quartile for percent reporting excessive drinking behaviors, but higher rates of alcohol-DWI suspension events occurred in counties in the lower quartiles of excessive drinking. Finally, more than 40% of initial and repeat suspensions occurred in counties within the highest quartile of the residential segregation index, indicating a higher geographical separation between Black and White residents in those counties. These counties also had the highest rate of overall suspension events. The secondary analysis of contextual characteristics associated with the actual duration of suspensions in terms of suspension-years (S2 Table) indicated similar patterns to the suspension event analysis.

### Trends in alcohol-DWI suspension events overall and by suspension type

**Fig 1:**
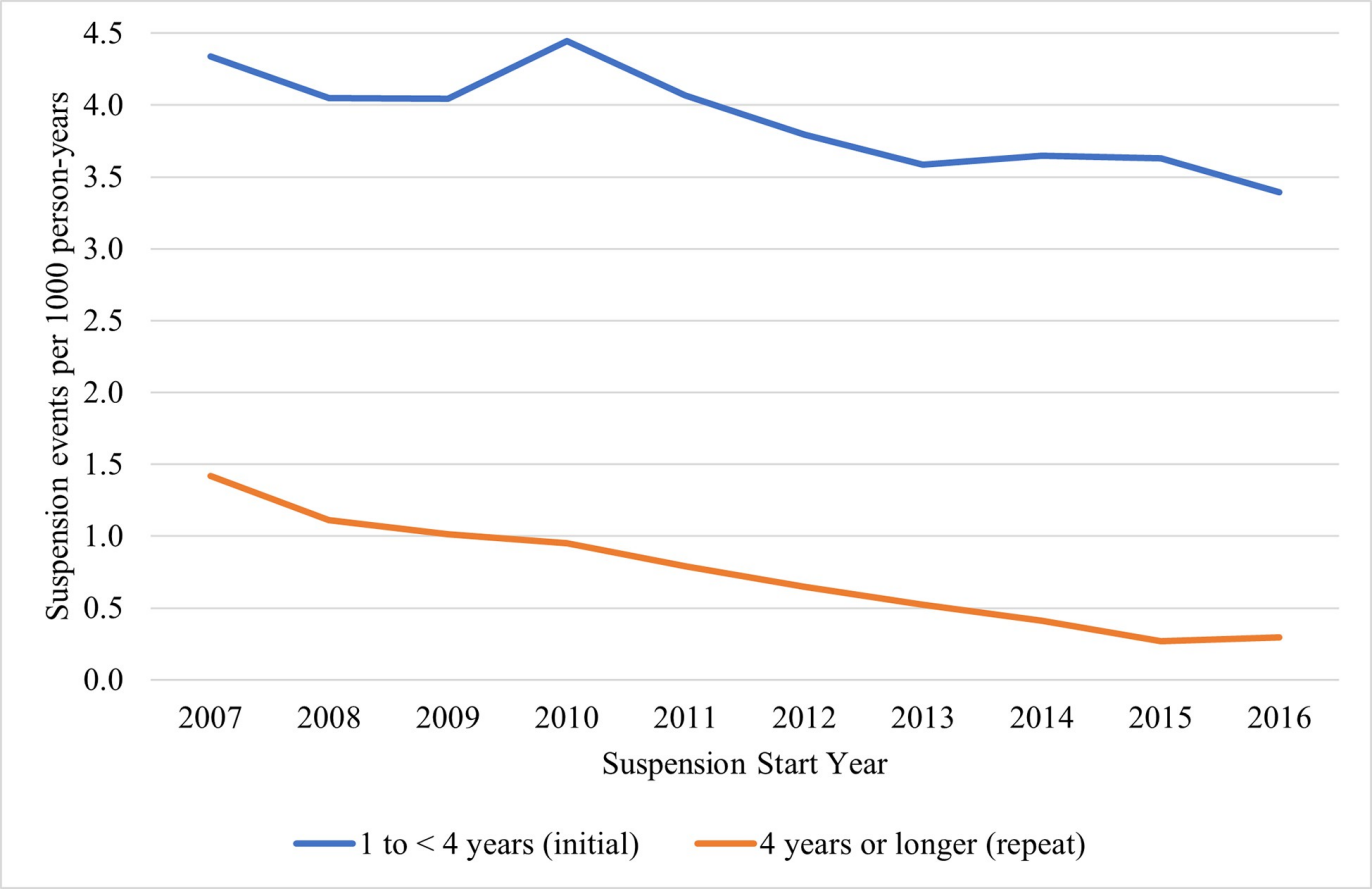
Rates of 1 year to <4 years (initial) and 4 years or longer (repeat) suspensions in North Carolina, 2007–2016.

Fig 1 displays changes in the annual alcohol-DWI suspension event rate over the 11-year study period overall and by suspension duration type. Overall, the rate of suspension events decreased from 5.8 suspension events per 1,000 population (95% CI: 5.7, 5.8) in 2007 to 3.7 (95% CI: 3.6, 3.7) in 2016, a 36.2% decline. During this time, larger declines were observed for repeat, as compared to initial, suspension events. The initial suspension rate declined from 4.3 (95% CI: 4.3, 4.4) to 3.4 (95% CI: 3.3, 3.4) per 1,000 population (24% decline), while repeat suspensions declined by 80%, from 1.4 (95% CI: 1.4, 1.4) to 0.29 (95% CI: 0.28, 0.31) events per 1,000 population. Rates and corresponding 95% CIs for overall, initial and repeat suspensions over time are in S3 Table.

### Trends in alcohol-DWI suspension events by suspension type and sex

**Fig 2:**
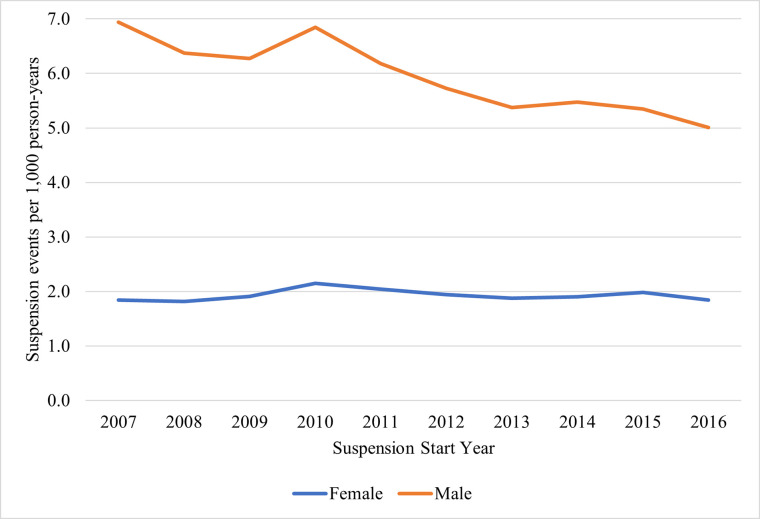
Rates of 1 year to <4 years (initial) suspensions by sex in North Carolina, 2007–2016.

For both initial and repeat suspensions, annual rates of suspension events were consistently higher among males than females across the study period (Fig 2 and S4 Table). Among males with initial suspensions, rates remained somewhat stable between 6.3 (95% CI: 6.2, 6.4) and 6.9 (95% CI: 6.8, 7.0) suspensions per 1,000 population between 2007 to 2010, followed by a consistent decrease to 5.0 per 1,000 population in 2016 (95% CI: 4.9, 5.1). However, females had a much different trend, with rates remaining stable across the entire study period.

**Fig 3:**
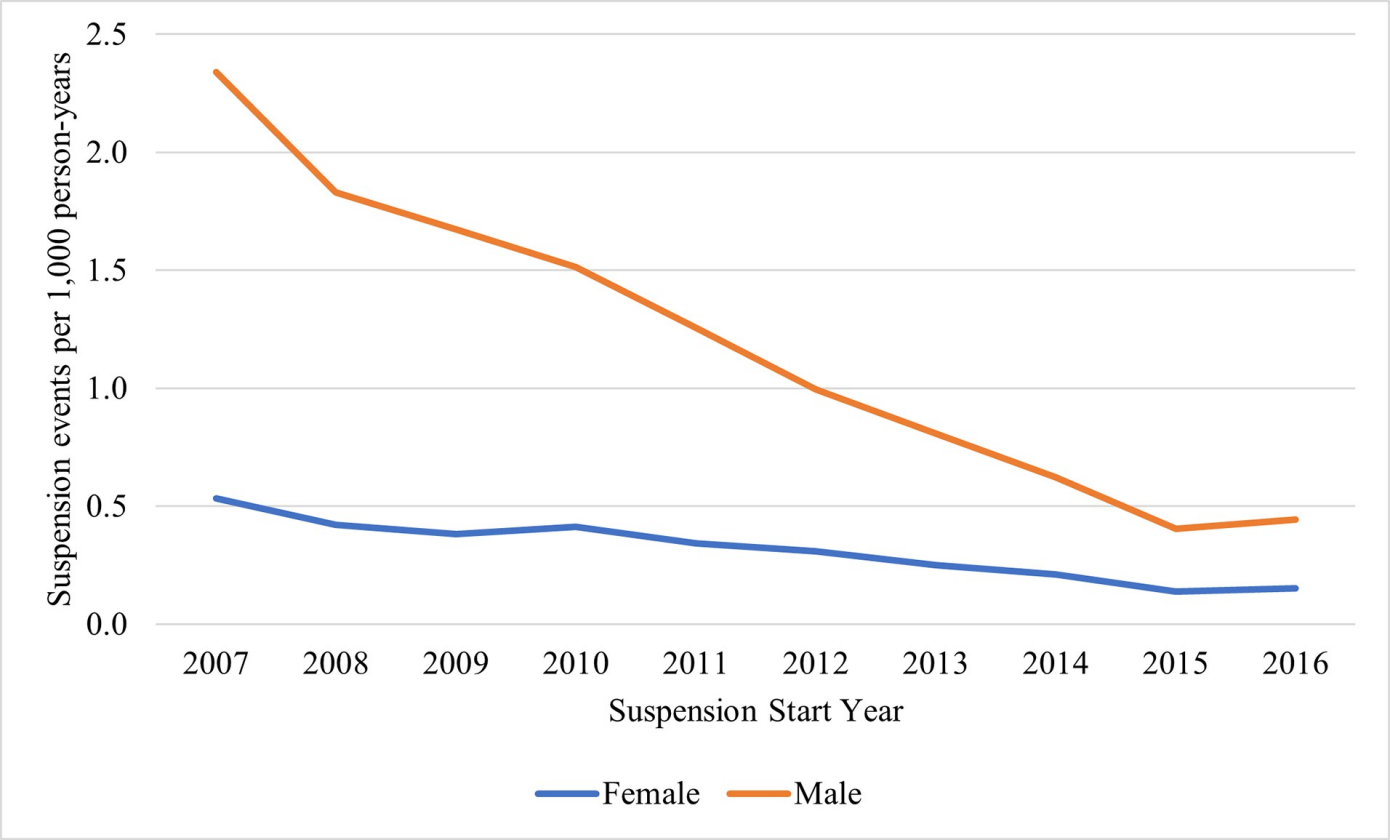
Rates of 4 years or longer (repeat) suspensions by sex in North Carolina, 2007–2016.

Among repeat suspension events, large decreases were observed among both males and females (Fig 3 and S4 Table). Among males, there was a substantial decrease from 2.3 (95% CI: 2.3, 2.4) in 2007 to 0.40 suspensions per 1,000 population (95% CI: 0.38, 0.43) in 2015 (83% decline), with rates stabilizing at 0.44 suspensions per 1,000 population (95% CI: 0.42, 0.47) in 2016. A similar, notable decrease was observed among females, 0.53 (95% CI: 0.51, 0.56) in 2007 to 0.14 (95% CI: 0.13, 0.15) in 2015 (80% decline), with a similar stabilization in 2016.

### Trends in alcohol-DWI suspension events by suspension type and race/ethnicity

**Fig 4:**
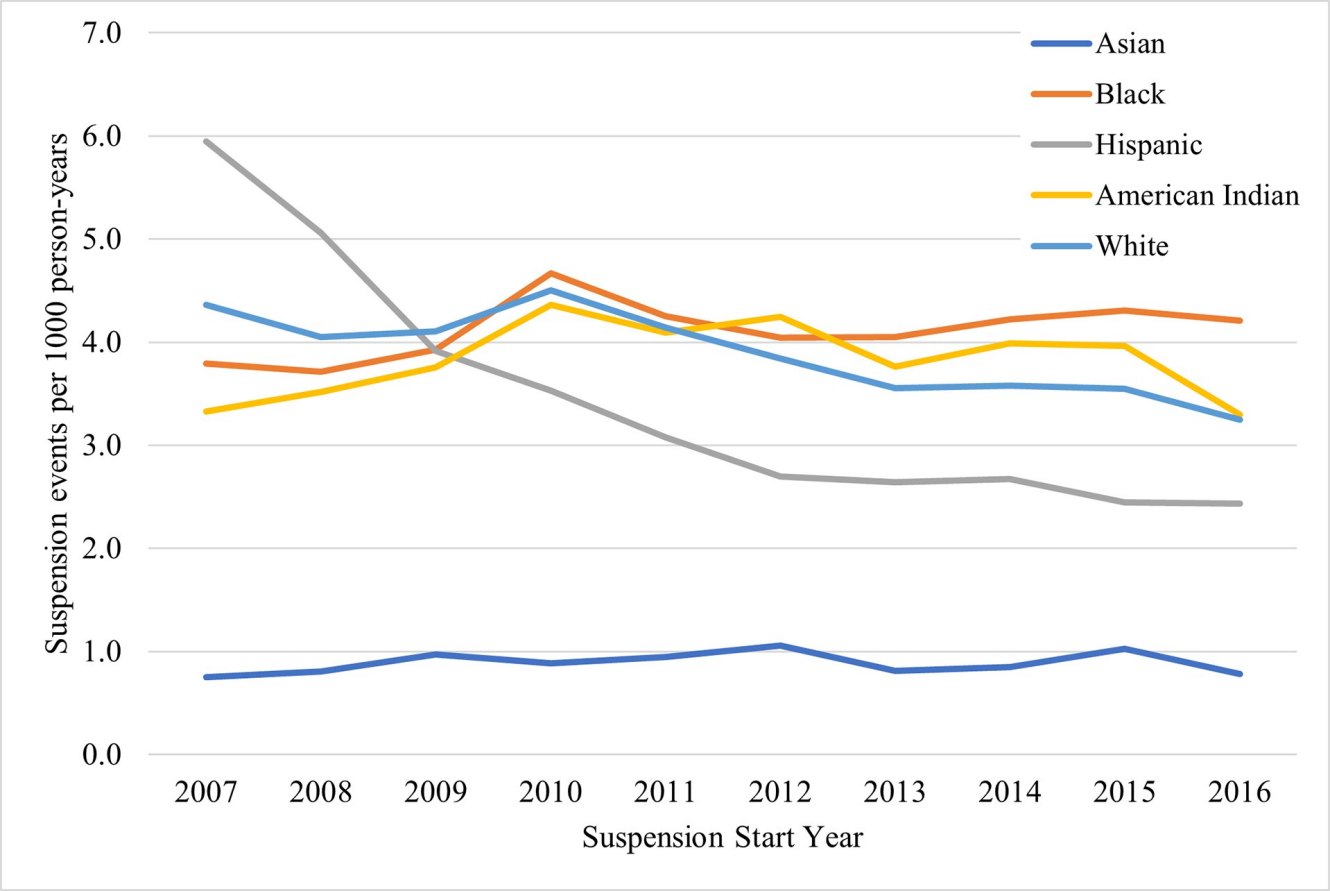
Rates of 1 year to <4 years (initial) suspensions by race/ethnicity in North Carolina, 2007–2016.

Among initial suspensions (Fig 4 and S5 Table), the largest change in rates of suspension events over the study period was observed among Hispanic individuals, who had the highest rate of all racial/ethnic groups in 2007 (5.9 per 1,000 population; 95% CI: 5.7, 6.2), with a substantial decrease to 3.9 (95% CI: 3.7; 4.1) in 2009, followed by a more gradual decrease to 2.4 suspensions per 1,000 population (95% CI: 2.3, 2.6) in 2015 and 2016. Among White, Black, and American Indian individuals, rates increased from 2007 to 2010, where all three rates coalesced at around 4.5 suspensions per 1,000 population, followed by decreases between 2010 and 2016 for American Indian and White individuals, while the rate among Black individuals remained relatively stable. Asian individuals had the lowest rates of suspension relative to other race/ethnicity groups consistently across the study period. By 2016, the highest rate was among Black individuals, followed by American Indian, White, Hispanic, and Asian individuals.

**Fig 5:**
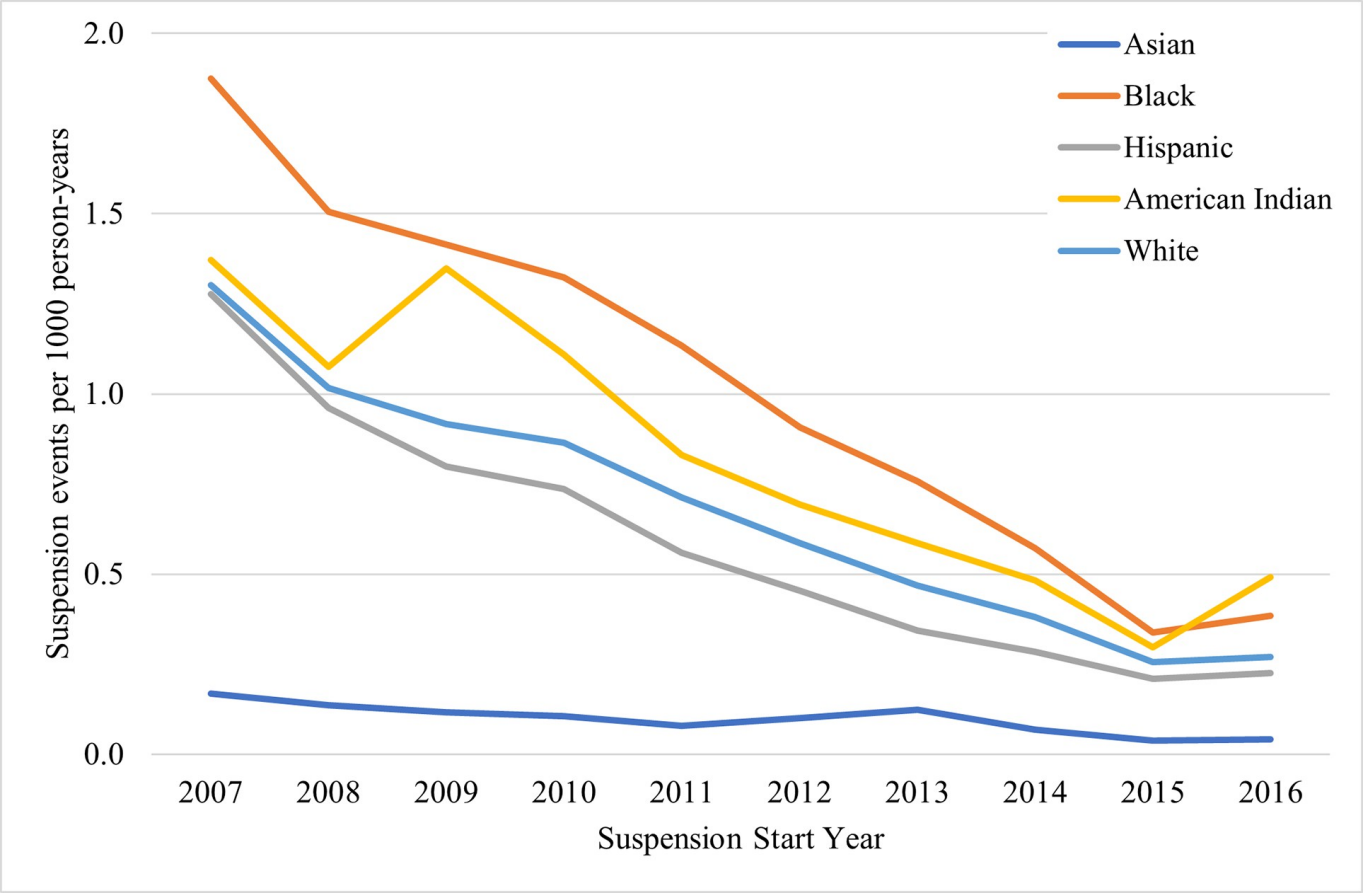
Rates of 4 years or longer (repeat) suspensions by race/ethnicity in North Carolina, 2007–2016.

Among repeat suspensions (Fig 5 and S5 Table), there were large suspension rate decreases across the study period in all racial and ethnic groups except among Asian individuals, whose rates remained consistently low. Black individuals had the highest rates from 2007 through 2015 (1.9 [95% CI: 1.8, 2.0] to 0.34 [95% CI: 0.31, 0.37] per 1,000 population), after which the rate among American Indian individuals became the highest at 0.49 events per 1,000 population (95% CI: 0.33, 0.65) in 2016.

## Discussion

Alcohol-DWI suspensions in NC are associated with several personal and contextual characteristics and have exhibited notable dynamics over time—providing critical information for informing future research and intervention efforts. First, we found that the rate of suspension events among males was over three times that of females, with differences even larger for repeat, as compared to initial, suspension events. However, trend analyses revealed decreases in these gaps over time, such that by 2016 males had initial and repeat suspension rates 2.8 and 2.0 times that of females, respectively. Second, we found that Black individuals had the highest rates of suspension events overall. While suspension rates declined across most races and ethnicities during the study period, and particularly for Hispanic individuals, initial suspension rates among Black and American Indian individuals remained relatively stable, slightly increasing between 2007 and 2016. Furthermore, Black individuals experienced notable differences in the average suspension-years served per 1,000 population, as compared to other races and ethnicities, indicating additional potential disparities in the actual duration of suspension events and potential barriers to ending a license suspension. Third and relatedly, contextual analyses indicated that the highest rates of suspension occurred in counties with high percentages of uninsured populations and the highest residential segregation index scores.

Our findings indicate a decrease in annual suspension rates, congruent with findings from nationwide data on DWI arrests and self-reported impaired driving [60, 61]; however, among drivers fatally injured in a crash, the proportion impaired remained around one-third [61]. Also consistent with prior research [1, 3–8, 60], we observed consistently higher alcohol-DWI suspension rates among males compared to females. However, contrary to patterns observed in research of fatal crashes [1], we observed overall decreases in suspension rates among both groups. Examination by suspension duration type, however, indicated decreases in both initial and repeat suspensions for males but only decreases in repeat suspensions for females. Trends revealed a narrowing gap between rates of license suspensions among males and females over the course of the study period–a pattern similarly observed in prior research [5, 7, 62, 63]. This prior research has hypothesized that narrowing gaps may be due to increasing trends of alcohol use among females, or changes in legal policies and enforcement practices that target more low-level offenders (frequently females) who were previously undetected [5, 7, 62, 63]. More recent research has examined underlying factors contributing to disparities by sex, such as impulsivity/lack of premeditation, criminal history, anxiety and other measures of physical and mental comorbidity [63–65].

Trends by race/ethnicity similarly revealed complex trends. While initial suspension rates among Black, American Indian, White and Asian individuals slightly increased between 2007 and 2010, we observed a large decrease in the annual suspension rate among Hispanic individuals, particularly between 2007 and 2012. Furthermore, we observed large decreases among most race/ethnicity groups over this time period for repeat suspensions. Changes in annual suspension rates can be influenced by a number of factors, including frequency of alcohol-impaired driving behaviors, enforcement practices, changes in population size/composition, or even changes in reported race/ethnicity information, which can impact numerator and denominator values in our rate calculations. Any one or combination of these factors may explain changes; however, to our knowledge, there were no major changes in race or ethnicity reporting or administrative database coding over this time period. Future research to understand more granular changes in potential enforcement practices, including number of events and focal enforcement locations, is warranted.

Detailed comparisons between our findings and prior research regarding race/ethnicity are difficult due to varying definitions and categorizations of race and ethnicity, as well as restricted comparison groups (e.g., comparing White and Black individuals only), yet we can broadly discuss and highlight some overall similarities and differences. Our study findings indicated that Black and American Indian individuals had greater rates of suspension events and, notably, of average time spent suspended (i.e., suspension-years) than other race/ethnicity groups. This finding is consistent with general criminal-legal system research finding higher rates of arrest among Black and American Indian individuals, as compared to other individuals, [11, 12, 16, 42] as well as findings from prior research of NC traffic stops indicating that Black drivers were more likely to be stopped than White drivers [16]. The differences observed in our study may also be explained by findings of previous research, which describe how additional barriers for ending driver’s license suspensions and initiating subsequent reinstatement (e.g., fines, fees, courses) disproportionately burden certain populations, including Black individuals [42, 66, 67]. However, our study is the first to quantify suspension durations, finding that Black individuals tend to spend an average of 1.5 years with a suspended license for an initial suspension event, 15% longer than Asian individuals. Additional research is needed to understand how these disparities relate to actual underlying impaired driving behaviors in NC. Recent research from the southwestern U.S. found that over-representation in drug- and alcohol-related arrests among minority populations could not be attributed to greater average use of alcohol or drugs [68]. Racial/ethnic disparities in alcohol-DWIs and associated outcomes should continue to be examined as a necessary first step toward addressing potential biases and working towards more equitable transportation and criminal-legal systems.

Relatedly, contextual variables may provide indications of structural factors that can help understand mechanisms driving observed demographic differences and potential disparities. We found that for both initial and repeat suspension types, frequencies and rates of suspension varied by quartile of residential segregation, urban/rural status, and proportion of the population without health insurance. Notably, we observed that a majority of initial and repeat suspensions occurred in counties with the greatest levels of Black/White residential segregation, which also had the highest suspension rates compared to counties with lower levels of segregation. Although the relationship between residential segregation and alcohol-DWI license suspension has not been previously explored, prior research indicates that higher levels of residential segregation are associated with increased alcohol outlet density and increased sizes of police departments per resident population, while also contributing to other racial and ethnic disparities in SES, self-rated health, and access to healthcare [69–71]. Given our study findings and the complex relationships already established in prior literature, future research should prioritize examining the association between alcohol-DWIs and residential segregation. Intensity of commonly used strategies for reducing alcohol-impaired driving, such as sobriety checkpoints and high-visibility saturation patrols, could be directly influenced by residential segregation (via local police strength). Future research examining alignment with police department size and location and potential disparities in alcohol-DWI enforcement is warranted. Finally, we observed trends in suspension rates by rural/urban status and health insurance but little variation by unemployment and population living below the poverty limit. Continued examination of these relationships is warranted given mixed findings in prior research [5, 11, 19] and their relevance when exploring factors contributing to alcohol-DWIs from a socio-ecological perspective. Our study is novel in its inclusion of these important contextual variables, which can give us insight into potential underlying mechanisms and structural contributors to alcohol-DWI suspensions and associated disparities. It is also important to recognize that alcohol-DWI suspensions require a series of events to occur prior to license suspension (i.e., alcohol consumption, alcohol-impaired driving behavior, and identification by law enforcement), and that disparities exist along this pathway. In addition to including contextual factors, future studies should further examine potential disparities along the pathway from citation through conviction, examine other alcohol-DWI sanctions (e.g., mandatory breathalyzers, alcohol education programs), and include both time varying and non-time varying variables as predictors of trends.

### Limitations

Findings should be interpreted in light of limitations. First, since variables reflecting personal characteristics come from administrative licensing data, these analyses could only be completed for those with non-missing data. A similar problem arose when analyzing contextual characteristics, as these could only be evaluated for suspension events where county of residence, necessary for data linkage, was available. However, missingness was small (approximately 2%), and we would therefore not expect it to impact inferences regarding the context for alcohol-DWI suspensions. Second and relatedly, residential segregation index scores were not computed for counties with Black populations of less than 100 individuals in a given year. However, this only affected 10 counties (10%) in NC. Third, while contextual data allows us to take a more holistic look at potential underlying factors influencing alcohol-DWI suspensions, these variables were not available at the individual level and therefore had to be obtained from external data sources at the county level–the smallest geographic level available across datasets. As such, the county measures associated with a suspension event may not necessarily reflect the specific circumstances of that individual but is rather an approximation of their environment. Further, it’s important to note that the time frame could influence county-level data, as events such as economic recessions may impact measures. Finally, as discussed below, an analysis of race/ethnicity comes with some limitations and requires a clear understanding of how this is measured. In NC licensing data, race and ethnicity are combined into a single optional self-report measure, where individuals may select one option from a list or may refuse to answer. Given the use of this combined variable, we cannot disentangle disparities by race and by ethnicity; however, we believe this study makes important contributions to our understanding of potential disparities.

### Considerations for data systems and future research

Our findings, in conjunction with those from prior studies, reveal potential disparities in alcohol-DWI license suspensions, however, these studies are limited by the quality of the data available. We propose several data system considerations to support better future examination of inequities in and impacts of implemented alcohol-DWI enforcement and prevention strategies (Box 1). First, consistent use of independent measures of ‘Race’ and ‘Ethnicity’ is needed across all NC administrative data systems (e.g., licensing, crash, and court data). These measures reflect separate and complex social constructs that reflect cultural identity, traditions, and societal experiences, and individuals may identify with different combinations of race and ethnicity (e.g., Black Hispanic or Black Non-Hispanic). Thus, classifications requiring a single response to a combined variable (e.g., Black or Hispanic) may not accurately reflect an individual’s nuanced social and cultural experiences. Additionally, these measures should remain optional due to the sensitive nature of this information which may be used to perpetuate disparities [72–75]. Second, collection of ‘Gender’ in addition to ‘Sex’ (with options to select either ‘Male’ or ‘Female’ only) may be beneficial. ‘Gender’ better reflects gender identity and expression, as well as the context and cultural experience of individuals, and would allow for better monitoring of suspension rates and law enforcement interactions with individuals who identify with minority genders. Recommendations by the National Academies of Sciences, Engineering, and Medicine [76] include examples of how these measures may be structured to be inclusive of different gender identities and also allow individuals to indicate a preference to not answer.

**Box 1. Considerations for improving collection of race and ethnicity data in licensing and associated data systems**

• Collect ‘Race’ and ‘Ethnicity’ as two separate variables

• Use more inclusive and specific categories of ‘Race’ and ‘Ethnicity’, such as the classification schemes available in Death Record and US Census data

• Provide the option to select multiple values as they apply to the individual (e.g., selecting 2 categories for ‘Race’)

• In all data systems (licensing, court, and crash), ‘Race’ and ‘Ethnicity’ should be collected via self-report where possible, or next of kin if necessary.

• Collect ‘Gender’ in addition to ‘Sex’. ‘Gender’ reflects gender identity and expression, and is a more accurate indicator of cultural expectations.

• Categories for ‘Gender’ should be more inclusive and allow a free-text field. We recommend categories for ‘Gender’ and ‘Sex’ as described in a report by the National Academies of Sciences, Engineering, and Medicine

• All aforementioned variables should include options for ‘Don’t know’ and ‘Prefer not to answer’ and remain optional so individuals are not forced to provide information in order to obtain licensure, and given the sensitive nature and potential for misuse of this information.

Finally, understanding underlying factors contributing to these disparities is critical for ensuring road safety strategies are equitably applied and designed in a way to maximize public health outcomes. Therefore, future research on alcohol-DWI suspensions and impaired driving should go beyond focusing on only demographic characteristics, but also continue to include county-level contextual characteristics (i.e., residential segregation) to examine underlying structures and mechanisms from a socio-ecological perspective, which recognizes the importance of examining factors from multiple levels (e.g., individual, community) when evaluating outcomes such as alcohol-DWI suspensions [55]. Such research can provide a more detailed foundation for improving the placement of and types of intervention approaches implemented, including approaches that are less punitive in nature, more treatment focused, and closely consider potential inequitable barriers to program completion. One such example is New York City’s Driver Accountability Program, which takes a restorative justice approach with guided exercises and discussions as an alternative to fines, and has demonstrated overall positive impacts on participants with improvements in driving beliefs and habits [77, 78].

### Conclusions

We found noteworthy differences in alcohol-DWI suspension trends by sex, race/ethnic group, and suspension type in NC between 2007 and 2016. Decreases were observed among males in both suspension types, while only among repeat suspensions for females. Among initial suspensions, Hispanic individuals demonstrated the largest decreases; however, we found little change in and persistently high rates among Black and American Indian populations. Notably, underlying contextual characteristics were also associated with varying suspension rates, including measures of Black/White residential segregation and percentage of population without health insurance. Our results contribute to an improved understanding of alcohol-DWI license suspensions in NC by exploring this issue from an arrest and court perspective and identifying differential impacts of suspensions on particular subgroups. Further, we considered our results, along with prior literature, to propose considerations for improving data systems to support future research on potential disparities in alcohol-DWI convictions and license suspensions, as well as inform future efforts to prevent alcohol-DWI in an equitable manner.

## Supporting information

S1 Table Rate of suspension-years by personal characteristics of drivers with alcohol-DWI suspensions in North Carolina, 2007–2016. (PDF)

S2 Table Rate of suspension-years contextual/county-level characteristics of drivers with alcohol-DWI suspensions in North Carolina, 2007–2016. (PDF)

S3 Table Annual rates of total, 1 year to <4 years (initial), and 4 years or longer (repeat) suspensions in North Carolina, 2007–2016. (PDF)

S4 Table Annual rates of 1 year to <4 years (initial) and 4 years or longer (repeat) suspensions by sex in North Carolina, 2007–2016. (PDF)

S5 Table Annual rates of 1 year to <4 years (initial) and 4 years or longer (repeat) suspensions by race/ethnicity in North Carolina, 2007–2016. (PDF)
