# Supplementary material for: Trends and disparities in alcohol-DWI license suspensions by suspension duration, North Carolina, 2007–2016
Source: PLoS One. 2024 Sep 20;19(9):e0310270. doi: 10.1371/journal.pone.0310270 (PMC11414890; doi:10.1371/journal.pone.0310270)
Supplement: S1 Table — (PDF) [file pone.0310270.s001.pdf]

S1 Table. Rate of suspension-years by personal characteristics of drivers* with alcohol-DWI suspensions in North Carolina, 2007 - 2016

	Total Suspension Events			Suspension Duration 1 year to <4 years (proxy for initial suspension)			Suspension Duration 4 years or longer (proxy for repeat suspension)		
	**Total no. of years suspended**	**% of years suspended**	**Rate of suspension-years per 1,000 person-years (95% CI)**	**Total no. of years suspended**	**% of years suspended**	**Rate of suspension-years per 1,000 person-years (95% CI)**	**Total no. of years suspended**	**% of years suspended**	**Rate of suspension-years per 1,000 person-years (95% CI)**
**Total**	552.268		9.7 (9.7, 9.8)	305,606	55.3	5.4 (5.4, 5.4)	246,662	44.7	4.4 (4.3, 4.4)
**Sex**									
**Female**	128,473	23.3	4.4 (4.4, 4.5)	76,517	25.0	2.6 (2.6, 2.7)	51,957	21.1	1.8 (1.8, 1.8)
**Male**	423,731	76.7	15.3 (15.2, 15.3)	229,069	75.0	8.3 (8.2, 8.3)	194,663	78.9	7.0 (7.0, 7.1)
**Race/ethnicity**									
**Asian**	2,686	0.5	1.7 (1.6, 1.8)	1,856	0.6	1.2 (1.1, 1.2)	830	0.3	0.53 (0.49, 0.56)
**Black**	150,913	27.8	12.3 (12.2, 12.3)	75,030	25.0	6.1 (6.0, 6.1)	75,884	31.3	6.2 (6.1, 6.2)
**Hispanic**	33,533	6.2	7.6 (7.5, 7.7)	19,189	6.4	4.4 (4.3, 4.4)	14,345	5.9	3.3 (3.2, 3.3)
**American Indian**	7,153	1.3	10.4 (10.2, 10.7)	3,729	1.2	5.4 (5.3, 5.6)	3,424	1.4	5.0 (4.8, 5.2)
**White**	348,196	64.2	9.2 (9.2, 9.3)	199,943	66.7	5.3 (5.3, 5.3)	148,253	61.1	3.9 (3.9, 4.0)
**Age at suspension**									
**21-24**	102,866	18.6	19.0 (18.9, 19.1)	54,569	17.9	10.1 (10.0, 10.2)	48,297	19.6	8.9 (8.9, 9.0)
**25-34**	194,615	35.2	15.4 (15.3, 15.4)	104,884	34.3	8.3 (8.2, 8.3)	89,731	36.4	7.1 (7.0, 7.1)
**35-44**	131,906	23.9	10.0 (10.0, 10.1)	70,995	23.2	5.4 (5.3, 5.4)	60,911	24.7	4.6 (4.6, 4.7)
**45-54**	91,714	16.6	6.7 (6.7, 6.8)	53,772	17.6	3.9 (3.9, 4.0)	37,942	15.4	2.8 (2.8, 2.8)
**55-64**	31,168	5.6	2.6 (2.6, 2.7)	21,386	7.0	1.8 (1.8, 1.8)	9,782	4.0	0.83 (0.81, 0.85)

** Note: Drivers may appear more than once in the table if they had multiple suspension events during the study period.*
Sex – based on self-report and required for licensing data; missingness <0.01%
Race/ethnicity – based on self-report where individuals may select one designation and optional for licensing data; missingness = 1.94%
Age at suspension – calculated using date of birth (required for licensing data) and start date of suspension event; missingness = 0.00%
