# Supplementary material for: Trends and disparities in alcohol-DWI license suspensions by suspension duration, North Carolina, 2007–2016
Source: PLoS One. 2024 Sep 20;19(9):e0310270. doi: 10.1371/journal.pone.0310270 (PMC11414890; doi:10.1371/journal.pone.0310270)
Supplement: S2 Table — (PDF) [file pone.0310270.s002.pdf]

S2 Table. Rate of suspension-years contextual/county-level characteristics of drivers with alcohol-DWI suspensions in North Carolina, 2007 - 2016

	**Total Suspension Events**			**Suspension Duration 1 year to <4 years (proxy for initial suspension)**			**Suspension Duration 4 years or longer (proxy for repeat suspension)**		
	**Total no. of years suspended**	**% of years suspended**	**Rate of suspension-years per 1,000 person-years (95% CI)**	**Total no. of years suspended**	**% of years suspended**	**Rate of suspension-years per 1,000 person-years (95% CI)**	**Total no. of years suspended**	**% of years suspended**	**Rate of suspension-years per 1,000 person-years (95% CI)**
**Urban/rural**									
**Metro**	382,961	71.4	9.1 (9.1, 9.1)	217,046	72.5	5.2 (5.1, 5.2)	165,915	70.0	3.9 (3.9, 4.0)
**Urban 20K or more**	91,387	17.0	11.0 (11.0, 11.1)	48,482	16.2	5.9 (5.8, 5.9)	42,906	18.1	5.2 (5.1, 5.2)
**Urban 2.5K - 19,999**	45,373	8.5	10.1 (10.0, 10.2)	24,841	8.3	5.5 (5.5, 5.6)	20,532	8.7	4.6 (4.5, 4.6)
**Rural**	16,662	3.1	10.0 (9.9, 10.2)	8,900	3.0	5.4 (5.3, 5.5)	7,762	3.3	4.7 (4.6, 4.8)
**Uninsured**									
**Quartile 1: < 14.49%**	151,242	28.2	8.4 (8.4, 8.5)	86,529	28.9	4.8 (4.8, 4.9)	64,712	27.3	3.6 (3.6, 3.6)
**Quartile 2: 14.49% to <15.69%**	185,888	34.7	10.4 (10.4, 10.5)	102,450	34.2	5.8 (5.7, 5.8)	83,438	35.2	4.7 (4.7, 4.7)
**Quartile 3: 15.69% to <17.35%**	142,218	26.5	9.1 (9.1, 9.2)	79,760	26.7	5.1 (5.1, 5.2)	62,458	26.3	4.0 (4.0, 4.0)
**Quartile 4: ≥ 17.35%**	57,035	10.6	11.1 (11.0, 11.2)	30,528	10.2	5.9 (5.9, 6.0)	26,507	11.2	5.1 (5.1, 5.2)
**Unemployed**									
**Quartile 1: < 9.09%**	165,401	30.8	9.2 (9.2, 9.3)	95,386	31.9	5.3 (5.3, 5.4)	70,015	29.5	3.9 (3.9, 3.9)
**Quartile 2: 9.09% to <10.71%**	161,969	30.2	8.9 (8.9, 9.0)	92,267	30.8	5.1 (5.1, 5.1)	69,702	29.4	3.8 (3.8, 3.9)
**Quartile 3: 10.71% to <11.91%**	119,733	22.3	10.2 (10.1, 10.2)	64,583	21.6	5.5 (5.4, 5.5)	55,150	23.3	4.7 (4.6, 4.7)
**Quartile 4: ≥ 11.91%**	89,280	16.6	10.4 (10.3, 10.4)	47,032	15.7	5.5 (5.4, 5.5)	42,249	17.8	4.9 (4.9, 5.0)
**Poverty**									
**Quartile 1: < 15.99%**	221,641	41.3	8.8 (8.8, 8.8)	128,124	42.8	5.1 (5.1, 5.1)	93,516	39.4	3.7 (3.7, 3.7)
**Quartile 2: 15.99% to <18.64%**	168,566	31.4	9.9 (9.9, 10.0)	92,872	31.0	5.5 (5.4, 5.5)	75,694	31.9	4.5 (4.4, 4.5)
**Quartile 3: 18.64% to <22.63%**	75,004	14.0	10.1 (10.0, 10.1)	40,416	13.5	5.4 (5.4, 5.5)	34,588	14.6	4.6 (4.6, 4.7)
**Quartile 4: ≥ 22.63%**	71,172	13.3	10.5 (10.4, 10.5)	37,856	12.6	5.6 (5.5, 5.6)	33,316	14.1	4.9 (4.8, 5.0)
**Health care access (Ratio of Pop to PCP)**									
**Quartile 1: < 1347.4**	253,108	47.2	8.8 (8.7, 8.8)	144,774	48.4	5.0 (5.0, 5.0)	108,334	45.7	3.8 (3.7, 3.8)
**Quartile 2: 1347.4 to <1988.4**	119,438	22.3	10.3 (10.3, 10.4)	65,048	21.7	5.6 (5.6, 5.7)	54,390	22.9	4.7 (4.7, 4.8)
**Quartile 3: 1988.4 to <2951.0**	99,718	18.6	10.5 (10.4, 10.5)	53,992	18.0	5.7 (5.6, 5.7)	45,726	19.3	4.8 (4.7, 4.8)
**Quartile 4: ≥ 2951.0**	64,119	12.0	9.8 (9.8, 9.9)	35,454	11.8	5.4 (5.4, 5.5)	28,664	12.1	4.4 (4.4, 4.5)
**Excessive drinking**									
**Quartile 1: < 9.9%**	59,742	11.1	10.5 (10.4, 10.6)	31,711	10.6	5.6 (5.5, 5.6)	28,031	11.8	4.9 (4.9, 5.0)
**Quartile 2: 9.9% to <12.0%**	76,701	14.3	10.5 (10.4, 10.6)	41,521	13.9	5.7 (5.6, 5.7)	35,180	14.8	4.8 (4.8, 4.9)
**Quartile 3: 12.0% to <14.0%**	159,285	29.7	9.7 (9.6, 9.7)	87,709	29.3	5.3 (5.3, 5.4)	71,576	30.2	4.4 (4.3, 4.4)
**Quartile 4: ≥ 14.0%**	240,655	44.9	8.9 (8.9, 8.9)	138,327	46.2	5.1 (5.1, 5.1)	102,328	43.2	3.8 (3.8, 3.8)
**Racial segregation index**									

**Quartile 1: < 28**	62,572	11.9	9.4 (9.3, 9.5)	34,749	11.8	5.2 (5.2, 5.3)	27823	11.9	4.2 (4.1, 4.2)
**Quartile 2: 28 to <36**	81,621	15.5	9.7 (9.7, 9.8)	44,027	15.0	5.2 (5.2, 5.3)	37594	16.1	4.5 (4.4, 4.5)
**Quartile 3: 36 to <46**	163,474	31.0	9.0 (8.9, 9.0)	92,443	31.4	5.1 (5.0, 5.1)	71031	30.4	3.9 (3.9, 3.9)
**Quartile 4: ≥ 46**	220,150	41.7	9.9 (9.9, 9.9)	123,199	41.8	5.5 (5.5, 5.6)	96951	41.5	4.4 (4.3, 4.4)

Urban/Rural – from Rural-Urban Continuum Codes (RUCC); missingness = 2.02% due to missing county information in suspension records
Uninsured, Unemployed, Poverty – from American Community Survey (ACS); missingness = 2.02% due to missing county information in suspension records
Health care access, Excessive drinking – from County Health Rankings (CHR); missingness = 2.02% due to missing county information in suspension records
Racial segregation – from County Health Rankings (CHR); missingness = 3.51% due to missing county information in suspension records and/or no residential segregation index score in CHR
Example interpretation for contextual variables: 28.2% of alcohol-DWI license suspension events occurred in counties falling in the lowest quartile of population uninsured, with an alcohol-DWI license rate of suspension-years in these counties of 9.1 per 1,000 person-years.
