# Supplementary material for: Trends and disparities in alcohol-DWI license suspensions by suspension duration, North Carolina, 2007–2016
Source: PLoS One. 2024 Sep 20;19(9):e0310270. doi: 10.1371/journal.pone.0310270 (PMC11414890; doi:10.1371/journal.pone.0310270)
Supplement: S3 Table — (PDF) [file pone.0310270.s003.pdf]

S3 Table. Annual rates of total, 1 year to <4 years (initial), and 4 years or longer (repeat) suspensions in North Carolina, 2007-2016

	Total Suspension Events			Suspension Duration 1 year to <4 years (proxy for initial suspension)			Suspension Duration 4 years or longer (proxy for repeat suspension)		
	**Total no. of suspension events**	**% of suspension events**	**Rate of suspension events per 1,000 person-years (95% CI)**	**Total no. of suspension events**	**% of suspension events**	**Rate of suspension events per 1,000 person-years (95% CI)**	**Total no. of suspension events**	**% of suspension events**	**Rate of suspension events per 1,000 person-years (95% CI)**
**2007**	30,970	11.8	5.8 (5.7, 5.8)	23,344	10.6	4.3 (4.3, 4.4)	7,626	18.4	1.4 (1.4, 1.4)
**2008**	28,283	10.8	5.2 (5.1, 5.2)	22,191	10.1	4.0 (4.0, 4.1)	6,092	14.7	1.1 (1.1, 1.1)
**2009**	28,099	10.7	5.1 (5.0, 5.1)	22,468	10.2	4.0 (4.0, 4.1)	5,631	13.6	1.0 (1.0, 1.0)
**2010**	30,227	11.5	5.4 (5.3, 5.5)	24,901	11.3	4.4 (4.4, 4.5)	5,326	12.8	1.0 (0.93, 1.0)
**2011**	27,574	10.5	4.9 (4.8, 4.9)	23,090	10.5	4.1 (4.0, 4.1)	4,484	10.8	0.79 (0.77, 0.81)
**2012**	25,348	9.7	4.4 (4.4, 4.5)	21,663	9.8	3.8 (3.7, 3.8))	3,685	8.9	0.65 (0.62, 0.67)
**2013**	23,605	9.0	4.1 (4.1, 4.2)	20,604	9.3	3.6 (3.5, 3.6)	3,001	7.2	0.52 (0.50, 0.54)
**2014**	23,491	9.0	4.1 (4.0, 4.1)	21,104	9.6	3.6 (3.6, 3.7)	2,387	5.7	0.41 (0.40, 0.43)
**2015**	22,721	8.7	3.9 (3.8, 3.9)	21,155	9.6	3.6 (3.6, 3.7)	1,566	3.8	0.27 (0.26, 0.28)
**2016**	21,679	8.3	3.7 (3.6, 3.7)	19,951	9.0	3.4 (3.3, 3.4)	1,728	4.2	0.29 (0.28, 0.31)
