# Supplementary material for: Trends and disparities in alcohol-DWI license suspensions by suspension duration, North Carolina, 2007–2016
Source: PLoS One. 2024 Sep 20;19(9):e0310270. doi: 10.1371/journal.pone.0310270 (PMC11414890; doi:10.1371/journal.pone.0310270)
Supplement: S4 Table — (PDF) [file pone.0310270.s004.pdf]

S4 Table. Annual rates of 1 year to <4 years (initial) and 4 years or longer (repeat) suspensions by sex in North Carolina, 2007-2016

	Suspension Duration 1 year to <4 years (proxy for initial suspension)			Suspension Duration 4 years or longer (proxy for repeat suspension)		
	**Total no. of suspension events**	**% of suspension events**	**Rate of suspension events per 1,000 person-years (95% CI)**	**Total no. of suspension events**	**% of suspension events**	**Rate of suspension events per 1,000 person-years (95% CI)**
**Females**						
**2007**	5067	9.1	1.8 (1.8, 1.9)	1462	16.2	0.53 (0.51, 0.56)
**2008**	5070	9.1	1.8 (1.8, 1.9)	1174	13.0	0.42 (0.40, 0.44)
**2009**	5423	9.7	1.9 (1.9, 2.0)	1084	12.0	0.38 (0.36, 0.40)
**2010**	6142	11.0	2.1 (2.1, 2.2)	1179	13.1	0.41 (0.39, 0.44)
**2011**	5927	10.6	2.0 (2.0, 2.1)	998	11.1	0.34 (0.32, 0.37)
**2012**	5675	10.2	1.9 (1.9, 2.0)	903	10.0	0.31 (0.29, 0.33)
**2013**	5508	9.9	1.9 (1.8, 1.9)	734	8.1	0.25 (0.23, 0.27)
**2014**	5620	10.1	1.9 (1.9, 2.0)	626	6.9	0.21 (0.20,0.23)
**2015**	5905	10.6	2.0 (1.9, 2.0)	414	4.6	0.14 (0.13, 0.15)
**2016**	5530	9.9	1.8 (1.8, 1.9)	453	5.0	0.15 (0.14, 0.16)
**Males**						
**2007**	18274	11.1	6.9 (6.8, 7.0)	6160	19.0	2.3 (2.3, 2.4)
**2008**	17118	10.4	6.4 (6.3, 6.5)	4918	15.1	1.8 (1.8, 1.9)
**2009**	17045	10.4	6.3 (6.2, 6.4)	4547	14.0	1.7 (1.6, 1.7)
**2010**	18757	11.4	6.8 (6.7, 6.9)	4146	12.8	1.5 (1.5, 1.6)
**2011**	17162	10.4	6.2 (6.1, 6.3)	3485	10.7	1.3 (1.2, 1.3)
**2012**	15987	9.7	5.7 (5.6, 5.8)	2781	8.6	1.0 (1.0, 1.0)
**2013**	15096	9.2	5.4 (5.3, 5.5)	2267	7.0	0.81 (0.77, 0.84)
**2014**	15484	9.4	5.5 (5.4, 5.6)	1761	5.4	0.62 (0.59, 0.65)
**2015**	15248	9.3	5.3 (5.3, 5.4)	1152	3.5	0.40 (0.38, 0.43)
**2016**	14418	8.8	5.0 (4.9, 5.1)	1274	3.9	0.44 (0.42, 0.47)
