# Supplementary material for: Trends and disparities in alcohol-DWI license suspensions by suspension duration, North Carolina, 2007–2016
Source: PLoS One. 2024 Sep 20;19(9):e0310270. doi: 10.1371/journal.pone.0310270 (PMC11414890; doi:10.1371/journal.pone.0310270)
Supplement: S5 Table — (PDF) [file pone.0310270.s005.pdf]

S5 Table. Annual rates of 1 year to <4 years (initial) and 4 years or longer (repeat) suspensions by race/ethnicity in North Carolina, 2007-2016

	Suspension Duration 1 year to <4 years (proxy for initial suspension)			Suspension Duration 4 years or longer (proxy for repeat suspension)		
	**Total no. of suspension events**	**% of suspension events**	**Rate of suspension events per 1,000 person-years (95% CI)**	**Total no. of suspension events**	**% of suspension events**	**Rate of suspension events per 1,000 person-years (95% CI)**
**Asian**						
**2007**	93	6.6	0.75 (0.60, 0.90)	21	14.4	0.17 (0.10, 0.24)
**2008**	106	7.6	0.80 (0.65, 1.0)	18	12.3	0.14 (0.07, 0.20)
**2009**	134	9.6	1.0 (0.81, 1.1)	16	11.0	0.12 (0.06, 0.17)
**2010**	126	9.0	0.88 (0.73, 1.0)	15	10.3	0.11 (0.05, 0.16)
**2011**	143	10.2	0.94 (0.79, 1.1)	12	8.2	0.08 (0.03, 0.12)
**2012**	169	12.1	1.1 (0.90, 1.2)	16	11.0	0.10 (0.05, 0.15)
**2013**	137	9.8	0.81 (0.68, 0.95)	21	14.4	0.12 (0.07, 0.18)
**2014**	150	10.7	0.85 (0.71, 1.0)	12	8.2	0.07 (0.03, 0.11)
**2015**	190	13.6	1.0 (0.88, 1.2)	7	4.8	0.04 (0.01, 0.07)
**2016**	153	10.9	0.78 (0.66, 0.90)	8	5.5	0.04 (0.01, 0.07)
**Black**						
**2007**	4299	8.5	3.8 (3.7, 3.9)	2127	17.3	1.9 (1.8, 2.0)
**2008**	4310	8.5	3.7 (3.6, 3.8)	1749	14.2	1.5 (1.4, 1.6)
**2009**	4651	9.2	3.9 (3.8, 4.0)	1676	13.6	1.4 (1.3, 1.5)
**2010**	5601	11.0	4.7 (4.5, 4.8)	1588	12.9	1.3 (1.3, 1.4)
**2011**	5218	10.3	4.2 (4.1, 4.4)	1393	11.3	1.1 (1.1, 1.2)
**2012**	5036	9.9	4.0 (3.9, 4.2)	1130	9.2	0.91 (0.85, 1.0)
**2013**	5129	10.1	4.0 (3.9, 4.2)	959	7.8	0.76 (0.71, 0.81)
**2014**	5422	10.7	4.2 (4.1, 4.3)	734	6.0	0.57 (0.53, 0.61)
**2015**	5590	11.0	4.3 (4.2, 4.4)	439	3.6	0.34 (0.31, 0.37)
**2016**	5527	10.9	4.2 (4.1, 4.3)	505	4.1	0.38 (0.35, 0.42)
**Hispanic**						
**2007**	2220	15.0	5.9 (5.7, 6.2)	477	19.4	1.3 (1.2, 1.4)
**2008**	2025	13.7	5.1 (4.8, 5.3)	384	15.6	1.0 (0.86, 1.1)
**2009**	1647	11.1	3.9 (3.7, 4.1)	336	13.6	0.80 (0.71, 0.88)
**2010**	1534	10.4	3.5 (3.4, 3.7)	320	13.0	0.74 (0.66, 0.82)
**2011**	1358	9.2	3.1 (2.9, 3.2)	247	10.0	0.56 (0.49, 0.63)
**2012**	1209	8.2	2.7 (2.5, 2.8)	203	8.2	0.45 (0.39, 0.52)
**2013**	1202	8.1	2.6 (2.5, 2.8)	156	6.3	0.34 (0.29, 0.40)
**2014**	1235	8.4	2.7 (2.5, 2.8)	131	5.3	0.28 (0.23, 0.33)
**2015**	1162	7.9	2.4 (2.3, 2.6)	99	4.0	0.21 (0.17, 0.25)
**2016**	1190	8.1	2.4 (2.3, 2.6)	110	4.5	0.23 (0.18, 0.27)
**American Indian**						
**2007**	216	8.2	3.3 (2.9, 3.8)	89	15.9	1.4 (1.1, 1.7)
**2008**	232	8.8	3.5 (3.1, 4.0)	71	12.7	1.1 (0.83, 1.3)
**2009**	251	9.5	3.8 (3.3, 4.2)	90	16.0	1.3 (1.1, 1.6)
**2010**	295	11.2	4.4 (3.9, 4.9)	75	13.4	1.1 (0.86, 1.4)
**2011**	281	10.7	4.1 (3.6, 4.6)	57	10.2	0.83 (0.61, 1.0)

**2012**	294	11.2	4.2 (3.8, 4.7)	48	8.6	0.69 (0.50, 0.89)
**2013**	263	10.0	3.8 (3.3, 4.2)	41	7.3	0.59 (0.41, 0.77)
**2014**	281	10.7	4.0 (3.5, 4.5)	34	6.1	0.48 (0.32, 0.64)
**2015**	281	10.7	4.0 (3.5, 4.4)	21	3.7	0.30 (0.17, 0.42)
**2016**	235	8.9	3.3 (2.9, 3.7)	35	6.2	0.49 (0.33, 0.65)
**White**						
**2007**	16064	11.0	4.4 (4.3, 4.4)	4791	18.9	1.3 (1.3, 1.3)
**2008**	15079	10.3	4.1 (4.0, 4.1)	3785	14.9	1.0 (1.0, 1.0)
**2009**	15359	10.5	4.1 (4.0, 4.2)	3433	13.5	0.92 (0.89, 0.95)
**2010**	16909	11.5	4.5 (4.4, 4.6)	3246	12.8	0.86 (0.83, 0.89)
**2011**	15680	10.7	4.1 (4.1, 4.2)	2703	10.6	0.71 (0.69, 0.74)
**2012**	14531	9.9	3.8 (3.8, 3.9)	2217	8.7	0.59 (0.56, 0.61)
**2013**	13452	9.2	3.6 (3.5, 3.6)	1771	7.0	0.47 (0.45, 0.49)
**2014**	13565	9.3	3.6 (3.5, 3.6)	1441	5.7	0.38 (0.36, 0.40)
**2015**	13463	9.2	3.5 (3.5, 3.6)	969	3.8	0.26 (0.24, 0.27)
**2016**	12378	8.5	3.2 (3.2, 3.3)	1029	4.1	0.27 (0.25, 0.29)
